# Post Arrest Consult Team: a knowledge translation strategy for post-cardiac arrest care

**DOI:** 10.1186/cc13682

**Published:** 2014-03-17

**Authors:** SC Brooks, D Scales, K Dainty, S Gray, R Pinto, E Racz, M Gaudio, A Amaral, A Baker, M Chapman, E Crystal, P Dorian, N Fam, R Fowler, J Friedrich, M Madan, G Rubenfeld, O Smith, LJ Morrison

**Affiliations:** 1 Queen's University, Kingston, Canada; 2 University of Toronto, Canada; 3 Sunnybrook Health Sciences Centre, Toronto, Canada; 4 St Michael's, Toronto, Canada

## Introduction

Lack of standardized care contributes to low survival in admitted out-of-hospital cardiac arrest (OHCA) patients. The objective of our study was to implement a Post Arrest Consult Team (PACT) and improve the quality of care for admitted OHCA patients.

## Methods

We conducted a prospective cohort study with concurrent controls from February 2011 to February 2013 in a network of 29 Toronto-area hospitals. The PACT was implemented in two hospitals and functioned as a consult service with a nurse and physician on- call 24/7. Patients from other network hospitals acted as concurrent controls. The PACT focused on four key processes of care: targeted temperature management (TTM); coronary angiography; avoidance of premature withdrawal of life-sustaining therapy (WLST <72 hours) on the basis of neuroprognostication; and electrophysiology assessment. We included nontraumatic OHCA patients who were >18 years old, survived at least 6 hours, and were comatose. We excluded patients with do-not-resuscitate orders, intracranial or other severe bleeding. We used generalized linear mixed models to assess whether PACT implementation was associated with higher odds of achieving each of the four targeted processes of care while adjusting for secular trends unrelated to the intervention.

## Results

The primary analysis included 162 patients from two intervention hospitals and 892 from 27 control hospitals. Thirty- two percent of the patients were female and the mean age was 65.3 ± 16.5 years. Almost one-half (46%) of patients had a shockable initial cardiac arrest rhythm, 41% had bystander CPR, and 5% had an AED applied. PACT did not improve use of TTM (ratio of ORs = 1.03, 95% CI = 0.89 to 1.20), angiography for patients without ST-elevation myocardial infarction (ratio of ORs = 1.10, 95% CI = 0.87 to 1.40), or electrophysiology assessment (ratio of ORs = 1.06, 95% CI = 0.81 to 1.38) as compared with concurrent control hospitals. Patients in the intervention group were less likely to have life support withdrawn within 72 hours on the basis of neuroprognosis compared with patients in the concurrent control group (ratio of ORs = 0.62, 95% CI = 0.39 to 0.98).

## Conclusion

PACT was associated with reduced WLST <72 on the basis of neuroprognostication but did not improve other important post-cardiac arrest processes of care. Further work is underway to identify factors that influenced implementation. This will guide future consideration of the PACT model in other settings.

